# Towards an African-led model for strengthening capacity in medical statistics and epidemiology in sub-Saharan Africa: an equitable partnership approach

**DOI:** 10.1093/ije/dyag006

**Published:** 2026-02-07

**Authors:** Miriam Abdulla, Nuredin Mohammed, Tobias Chirwa, Andrew Abaasa, Sian Floyd, Emily L Webb, Philip Ayieko, Victoria Simms, Elizabeth C George, Thomas Gachie, Francis Kiroro, Helen A Weiss

**Affiliations:** International Statistics and Epidemiology Group, Faculty of Epidemiology and Population Health, London School of Hygiene and Tropical Medicine, London, United Kingdom; Medical Research Council Unit, The Gambia at London School of Hygiene & Tropical Medicine, Banjul, The Gambia; School of Public Health, Faculty of Health Sciences, University of the Witwatersrand, Johannesburg, South Africa; Medical Research Council/Uganda Virus Research Institute & London School of Hygiene and Tropical Medicine, Uganda Virus Research Institute, Entebbe, Uganda; International Statistics and Epidemiology Group, Faculty of Epidemiology and Population Health, London School of Hygiene and Tropical Medicine, London, United Kingdom; International Statistics and Epidemiology Group, Faculty of Epidemiology and Population Health, London School of Hygiene and Tropical Medicine, London, United Kingdom; International Statistics and Epidemiology Group, Faculty of Epidemiology and Population Health, London School of Hygiene and Tropical Medicine, London, United Kingdom; Mwanza Intervention Trials Unit, National Institute for Medical Research, Mwanza, Tanzania; International Statistics and Epidemiology Group, Faculty of Epidemiology and Population Health, London School of Hygiene and Tropical Medicine, London, United Kingdom; The Health Research Unit Zimbabwe, Biomedical Research and Training Institute, Harare, Zimbabwe; MRC Clinical Trials Unit at UCL, University College London, London, United Kingdom; International Statistics and Epidemiology Group, Faculty of Epidemiology and Population Health, London School of Hygiene and Tropical Medicine, London, United Kingdom; Data Synergy and Evaluations, African Population and Health Research Center, Nairobi, Kenya; International Statistics and Epidemiology Group, Faculty of Epidemiology and Population Health, London School of Hygiene and Tropical Medicine, London, United Kingdom

The demand for expertise in applied medical statistics and epidemiology in Africa exceeds the supply [1, 2] and it is rapidly increasing, with large-scale data sources such as electronic health records, bioimaging, genomics, social media, geospatial data, mobile phones, and wearable technology. In parallel, the expansion of statistical methods and tools (such as machine learning) means that it is essential to increase the number of people who are trained to analyse available data and stay abreast of methodological and technological advances for improving health in Africa. Rigorous study design and analysis are essential elements of good research and require well-trained, experienced statisticians who are integrated into research teams; a common misconception is that statisticians are only needed to analyse data rather than being an integral part of the research process.

Existing initiatives to increase capacity in medical statistics and health data science in Africa include the Wellcome-funded Sub-Saharan Africa Consortium for Advanced Biostatistics (SSACAB) and the International Biometrics Society Sub-Saharan African Network (IBS-SUSAN). SSACAB is a collaborative initiative uniting 4 African research institutions and 11 universities, supported by 4 European universities, with the aim of increasing the critical mass and promoting skills exchange in medical statistics methodology [3]. IBS-SUSAN is an African-led biometric network aiming to increase the application of statistics for advancing life sciences within the larger International Biometrics Society network.

To date, existing initiatives to increase capacity in medical statistics in Africa have primarily focused on Master of Science (MSc)- and Doctor of Philosophy (PhD)-level training programmes or bilateral partnerships between Global North and African institutions. Here, we describe the International Statistics and Epidemiology Partnership (ISEP), initiated in January 2024 through a 5-year UK Medical Research Council (MRC) partnership grant, which seeks to address a gap by supporting the entire career-development pathway of medical statisticians [1, 2].

## MRC International Statistics and Epidemiology Partnership (ISEP)

The goal of ISEP is to improve health in Africa by ensuring the high-quality design, analysis, and interpretation of epidemiological studies and to enhance capacity for surveillance and a timely response to current and emerging health threats. The aim of ISEP is to implement a sustainable strategy to strengthen capacity in applied medical statistics in Africa across the early and mid-career stages and to enable a pathway to be African-led by 2028.

The objectives are to:

- Create a network of medical statisticians (‘ISEP statisticians’) who lead quantitative aspects of epidemiological research in Africa. We will do this by providing a collaborative environment for knowledge sharing and continued professional development through peer support, mentoring, placements, and technical and transferable skills training.
- Increase the knowledge of medical statistics for global health research by sharing and consolidating statistical training material between ISEP statisticians and delivering training packages to postgraduate students and other biomedical researchers in Africa.
- Raise awareness of the need for increased capacity in medical statistics and contribute to the career pipeline for developing this capacity by initiating activities with policymakers, research leaders, and the youth.

**Figure 1.**
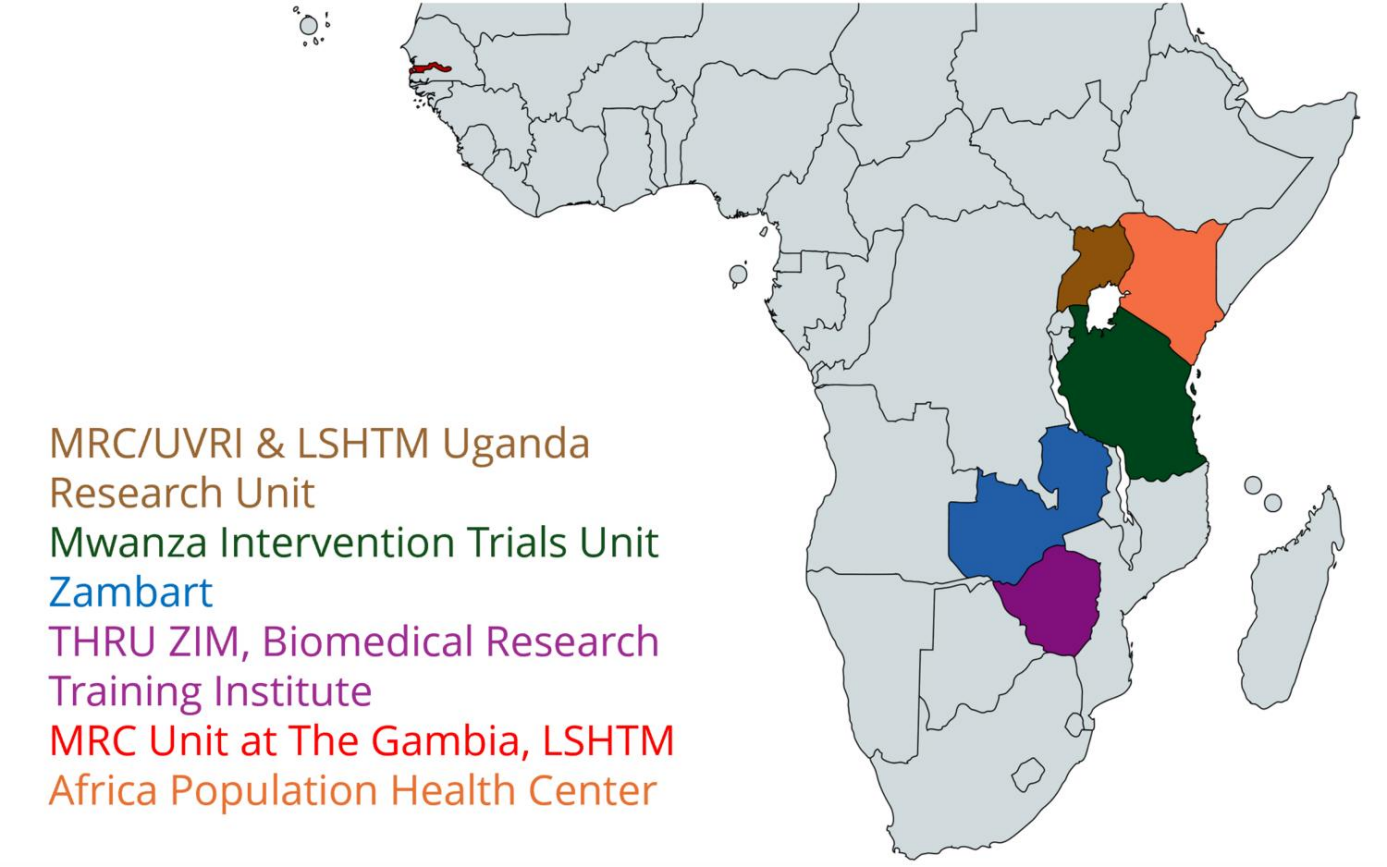
MRC ISEP African research institutes.

ISEP is a collaborative network of world-leading health research institutions—including six African research partner institutions and two UK institutions, each represented by a medical statistician co-investigator (Co-I). The research partner institutions are: the African Population and Health Research Center (APHRC) in Kenya, The Health Research Unit Zimbabwe (THRU ZIM); the Mwanza Intervention Trials Unit (MITU) in Tanzania; the MRC/UVRI & London School of Hygiene & Tropical Medicine (LSHTM) Uganda Research Unit (MRC/UVRI); MRC Unit The Gambia at LSHTM (MRC-G); Zambia AIDS Related Tuberculosis (Zambart), a research organization in Zambia; the MRC Clinical Trials Unit at University College London (UCL) London (MRC CTU); and LSHTM (Fig. 1). The expertise of each global health partner institution is shown in Table 1.

**Table 1.** Expertise at each of ISEP’s global health partner institutions.

Partner institution	Expertise
MRC-G	The research of the MRC-G at LSHTM is focused on vaccines and immunity, disease control and elimination, and nutrition and planetary health
MRC/UVRI and LSHTM Uganda Research Unit	The MRC/UVRI Unit conducts high-quality training and research for generating new knowledge and improving the control of infectious and non-communicable diseases in Africa and globally
MITU	MITU is an internationally recognized collaborative health research unit focusing on HIV and other related infections in Tanzania, with an emphasis on intervention trials
Zambart	Zambart is a research and capacity-strengthening institution based in Zambia working collaboratively with other research institutions, universities, and communities for improving public health. Zambart is a multidisciplinary team who conduct research at international, regional, national, and local levels for influencing policy and practice in HIV and tuberculosis and other critical public health issues
THRU ZIM	THRU ZIM conducts research in collaboration with local and international academic institutions and non-governmental organizations aimed at improving health across the lifespan in Zimbabwe and Africa
APHRC	APHRC is an African-led research institute conducting research on challenges facing Africa, including population, health, ageing, maternal and child health, and urbanization
MRC CTU	MRC CTU at UCL is Europe’s largest clinical trials unit and collaborates with multiple research institutes in Africa. The unit supports capacity strengthening through training and bursaries for short courses, MScs, and PhDs, and runs the Global Health Network website with free resources for researchers involved in clinical trials
LSHTM	LSHTM is the largest public health university in Europe, focusing on conducting research for improving health outcomes, reducing health inequities, and influencing policy and practice across the globe

HIV, human immunodeficiency virus.

In addition to the research partners, ISEP collaborates with strategic partners, namely: SSACAB; IBS-SUSAN; the African Research Excellence Fund [4] (AREF); Africa Centres for Disease Control and Prevention (CDC); and the African Institute for Mathematical Sciences (AIMS). The role of AREF is to mentor and support African scientists to participate equitably in health research to become role-model research experts and leaders. AREF prioritizes African-led partnership practices for ensuring high-quality, context-specific research for improving health in Africa. It also provides tailored technical and transferable skills courses for supporting the capacity strengthening of research and medical statistics and improved career development. Africa CDC strengthens the capacity of Africa’s public health institutions to detect and respond effectively to disease threats and outbreaks, based on data-driven interventions and programmes. Africa CDC and ISEP have a common goal of increasing the statistical capacity in sub-Saharan Africa (SSA) and will work together on joint grant applications, including those for statisticians to be based at Africa CDC. AIMS works to enable Africa’s early-career mathematicians in scientific training, research, and public engagement. AIMS will link students enrolled on its MSc in Mathematical Sciences course to African medical statisticians in ISEP, who will act as role models and provide supervision of their MSc projects.

## From the MRC International Statistics and Epidemiology Group to ISEP

We will achieve ISEP’s objectives by adapting a model established by the MRC International Statistics and Epidemiology Group (ISEG) at LSHTM [5]. ISEG was established in 1972 and focuses on the epidemiology and control of major public health problems of low- and middle- income countries (LMICs), predominantly in Africa. The core aims of ISEP are to develop research capacity in statistics and epidemiology, and achieve a health impact in LMICs. With programme grants from the MRC and project-specific funding, ISEG created and sustained a critical mass of applied statisticians and epidemiologists (34 as of October 2025), most of whom are UK-based. From 2003, ISEG implemented a 2-year MSc Fellowship scheme that enabled African science and maths graduates to undertake a 1-year MSc in Medical Statistics at LSHTM in London, followed by a 1-year placement at an African research institution linked to ISEG. In addition, ISEG led an initiative funded by the European and Developing Countries Clinical Trials Partnership (EDCTP), which supported African scientists to complete a 1-year MSc in Epidemiology at LSHTM and a short course on pandemic preparedness, response, and research, complemented by further training, networking, and dissemination activities [6]. Since 2003, 47 Fellows have completed one of these schemes and are employed as medical statisticians or epidemiologists in Africa (*n* = 34) and/or are undertaking or have undertaken PhDs (*n* = 25). A further 17 Fellows graduated from other EDCTP-funded biostatistics and epidemiology MSc schemes that were co-led by ISEG members.

ISEP draws on the ISEG model by creating a sustainable African-centred collaborative network of medical statisticians, to enable African medical statisticians to develop their skills and confidence, progress their careers, and forge a pathway to leadership of future research projects and capacity-strengthening initiatives [6]. There are strong links between ISEG and ISEP, as recent and current ISEG directors are principal investigators (PIs)/Co-Is for ISEP and two ISEG Fellows are Co-Is, ensuring continuity and the leverage of lessons learnt from ISEG.

## Needs assessment of capacity strengthening in medical statistics

ISEP was informed by the results of a survey circulated online to a wide network of medical statisticians and epidemiologists working with statisticians in global health research. The aim of the survey was to understand how to meaningfully and appropriately increase the medical statistics capacity in LMICs and retain medical statisticians in these countries. Solution-oriented questions were included to inform the design of ISEP as a strategy to overcome barriers faced by medical statisticians in Africa, focusing on mentorship and on-the-job training, formal training, networking, funding, job opportunities, and recruitment.

Sixty-two people responded to the survey, of whom 47 (76%) were based in Africa. The results are summarized in Table 2. Key findings from African respondents were the perceived lack of availability of mentors, limited formal training opportunities, lack of job opportunities, and lack of networking opportunities (58% of LMIC statisticians in the survey felt part of a network compared with 90% of those in high-income countries).

**Table 2.** Perceived barriers to a career in medical statistics.

Theme	Sub-theme	Perceived barriers to mentoring
Mentoring	Mentors	• Lack of time among potential mentors • Lack of mentors • Lack of hands-on training for mentorship
	Mentees	• Lack of knowledge on availability of mentoring programmes • Limited awareness of who currently is a mentor/can provide mentoring • Lack of knowledge of who to ask about mentorship opportunities/to mentor them
	Setting	• Mentorship programmes typically driven by high-income countries • Mentors tend to be from high-income countries • Few medical statisticians in many countries, especially in academia
	Institutional	• Lack of collaboration among medical statisticians • Lack of platform to link people for mentoring • Lack of funds for mentoring • Lack of suitable schemes
	**Theme**	**Perceived limitations of technical skills training**
Training	Content	• Too theoretical—needs to be more practical • Lack of real-world data • In LMIC countries, training courses for statisticians do not usually steer people towards a career in public health • Training courses are too short/not detailed enough/too specific • Training focuses on outdated data-analysis methods
	Accessibility	• Cost/funding • Lack of statistical software that is accessible in LMICs^a^ • Difficult for statisticians to get time off work for training • Lack of resources
	Setting	• Training is not widely available to those in Africa • In LMIC countries, training courses for statisticians do not usually steer people towards a career in public health
	**Theme**	**Perceived difficulties in recruiting medical statisticians**
Job opportunities/recruitment	Funding/salaries	• Salaries not competitive with jobs outside academia • LMICs lack funds to invest in setting up such opportunities
	Availability	• Trained and experienced statisticians are often not available to contribute to projects with low budgets • Lack of qualified applicants (enough jobs, but not enough statisticians)
	Career opportunities	• Medical statisticians choose other industries (rather than public health or medical research) due to a lack of opportunities and relatively low salaries

Source: International Statistics and Epidemiology Group 50th Symposium survey results presentation 2022.

a This is changing with the increasing move to R as a freely available software package.

LMIC, low- and middle-income country.

Potential solutions identified by the survey participants were for:

- mentoring to be available at different career stages, with clear intended outcomes and designed to be mutually beneficial to mentors and mentees;
- formal technical training to ensure that medical statisticians have updated skills, practical training to improve their ability to work with real-world contexts and data, and training in transferable skills;
- increasing equitable recruitment of medical statisticians through advocating for structural solutions—especially the prioritization of opportunities for LMIC statisticians, inclusion of statisticians on recruitment panels, recognition that statisticians are a key and integral part of a project team, and removal of a PhD-qualification requirement.

A central theme was the need for more funding and networking opportunities to facilitate training, mentoring, peer support, and recruitment, with embedded equitable practices required to enable all these solutions.

A limitation of our approach is that we do not know the total number of people who received the survey. The views of the responders may not be representative of the survey audience; however, they align with recommendations by Wellcome-funded consortia: SSACAB and the Sub-Saharan African Network for TB/HIV Research Excellence (SANTHE) [7].

Following the symposium, ISEG co-ordinated seven research partner institutions to apply for funding to form a partnership to address these barriers and opportunities.

## Methodological framework for ISEP

**Figure 2.**
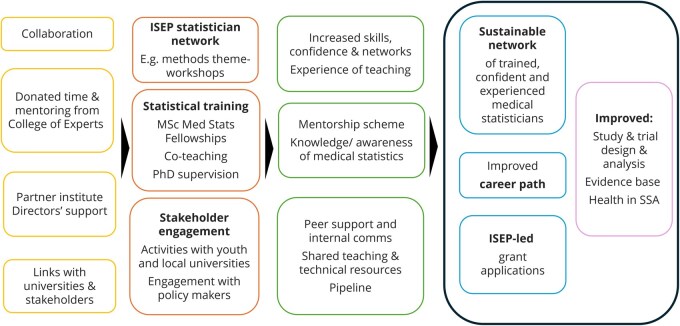
ISEP ToC diagram (simplified version). Left to right boxes: inputs; outputs; outcomes; short-term impacts; longer-term impact. (Work Package 1, WP1; Work Package 2, WP2; Work Package 3, WP3).

The Theory of Change (ToC) guides the partnership towards achieving its goals (Fig. 2).

Each component of the ToC draws upon theory, practical knowledge, and current evidence, including the ISEP survey and our partnership discussions. Our Monitoring and Evaluation (M&E) approach requires us to revisit the ToC periodically throughout the programme cycle and test the rationale and assumptions underpinning each ToC pathway and whether outcomes are being achieved in practice.

ISEP held an initiation workshop with our strategic partners at APHRC in Nairobi, Kenya in February and March 2024 to further understand which specific partnership activities are needed and how they are to be actioned and prioritized, to ensure that the needs of statisticians are met. There were 36 attendees: 17 African early- to mid-career medical statisticians (‘ISEP statisticians’) including 6 ISEP Co-Is, 4 Co-I/PIs from the UK, 6 representatives from strategic partners, and 3 ISEP co-ordinators. A total of 13 workshops were conducted by members of each of the six ISEP partner institutions, as well as its strategic partner, AREF. The first 3 days focused on ISEP planning, including how we might operationalize each Work Package (WP), followed by 3 days of technical and transferable skills training sessions.

## How we will achieve the objectives

ISEP WPs systematically address the barriers faced by medical statisticians in Africa, as follows:

### WP1: Facilitating ISEP statisticians to form a collaborative network

Through working with strategic partners, ISEP aims to improve the synergy between ISEP statisticians at different institutions and address the ‘brain drain’ (trained researchers from LMICs leaving their country of origin to work in high-income countries (HICs) highlighted in the EDCTP 2019 mid-term evaluation [8] by:

- holding monthly online themed workshops on statistical and epidemiological topics to facilitate knowledge exchange and technical skills strengthening;
- initiating a mentoring scheme tailored to ISEP statisticians to build mentee knowledge, capability, and self-reliance—the mentee is the primary driver of the relationship and the aim is to enable a two-way learning relationship for providing useful feedback and reflection opportunities for both mentee and mentor;
- forming cross-partnership working groups to work on specific projects such as the ISEP Equitable Partnerships Framework, grant proposals, and scientific papers;
- arranging specific transferrable skills training sessions run by AREF and other partners, including leadership and communication skills, and identifying and facilitating relevant technical training.

### WP2—Training in medical statistics

We are compiling a comprehensive list of courses offered by LSHTM, UCL and other relevant institutions to raise the awareness and accessibility of technical training options for prospective statisticians prior to MSc training. Two ISEP Co-Is established a short course for statisticians on how to deliver and facilitate R training, which took place in Zimbabwe in January 2025. The ISEP Applied Medical Statistics Fellowship scheme launched in January 2025, through which four African graduates undertake the 1-year MSc in Medical Statistics at LSHTM in London, UK, followed by a 1-year placement at an ISEP partner institute in Africa. ISEP received >500 applications for the MSc Fellowship in 2025. We will also facilitate PhD supervision and PhD student–supervisor matching, and adapt the ISEP mentoring scheme to support the aims of SSACAB and IBS-SUSAN.

### WP3—Stakeholder engagement

ISEP statisticians have had opportunities to connect with research leaders and other relevant stakeholders through conferences, including the National Institute for Health and Care Research Statistics Conference in Sheffield (UK) in June 2024. Other opportunities for stakeholder engagement have included the ISEP online launch event in October 2024, to which members of ISEP’s Scientific Advisory Committee were invited to offer their perspectives and experiences of being a statistician and solutions to strengthening career development through ISEP activities. The event attracted >100 attendees, with ∼40 joining the ISEP College of Experts. Two of these are from the Young African Statisticians Association (YASA)—an Africa-based organization aimed at supporting young African statisticians and demographers in their career development. ISEP currently collaborates with YASA on partnership activities through which there is mutual benefit, e.g. themed workshops. Future planned WP3 activities include further engagement with institution directors; engaging with local universities, AIMS, and policymakers; magazine features; and youth engagement, e.g. via schools outreach in partnership with YASA.

### WP4—Partnership management

Oversight, co-ordination and M&E are led by a full-time academic co-ordinator, supported by the Co-Is and administrative staff. Co-Is meet monthly online to discuss progress on action points. ISEP is overseen by an external Scientific Advisory Committee of seven senior scientists, mostly of African origin, with experience in medical research and partnership governance.

### Partnership equity

We have developed an Equitable Partnerships Framework to hold ourselves accountable to potential and/or existing inequities [9]. The framework was co-created by a working group of mixed gender, seniority, and location (Africa and the UK), and drew on existing equity toolkits and resources [10, 11]. To assess our evolving equitable practices and behaviours, we developed annual tools that include questions on individual and institutional power dynamics. ISEP considers ‘equitable partnership’ as an ‘ethical imperative’ [12] for inspiring a sense of community, commitment, and shared vision to achieving ISEP’s goals.

### Metrics for project success

Through our M&E framework, we will measure success against the outcomes in our ToC, via metrics detailed in an outcome framework. Metrics include the number of ISEP-led publications, short courses, workshops, and events. We will use a mixed-method evaluation approach to mitigate challenges around partnership operationalization [13] and limitations to achieving our desired outcomes and goals. For example, we will use qualitative metrics to assess how satisfied ISEP statisticians are with transferable skills training to provide context to the quantitative outcome ‘Increased transferable skills’. Data will be collected through annual online surveys and interviews in 2026 as part of a mid-term evaluation and at the end of 2028 for an end-of-programme evaluation.

## Conclusion

Sustainability of the network is a key future aim for ISEP. Our equitable partnerships approach and the continued development and application of its framework over time will orient us towards this aim. Retaining talented and well-trained medical statisticians in Africa is fundamental to ensuring ISEP’s sustainability. Africa-based leaders in medical statistics can inspire and train the next generation of medical statisticians. ISEP is on the path to achieving this goal through listening to the needs and wishes of Africa-based medical statisticians and accordingly tailoring training courses, mentorship schemes, and other learning and development opportunities including networking for their future success. Research-capacity-strengthening networks and equitable partnership work alone are not enough to sustain and grow the network. We plan to include additional initiatives to support ISEP’s growth and contribution to capacity strengthening in applied medical statistics beyond the end of the current grant period, including: (i) submitting grant proposals to involve youth in research design to provide pre-university and university students with insights into a medical research career [14]; (ii) using evidence from our evaluation reports to leverage further funding—specifically from Africa-based funders to retain talent in Africa; (iii) using research and evaluation evidence to influence policies in the UK and Africa for shifting funds from UK/global north ownership to African ownership; (iv) building interdisciplinary partnerships and networks based in Africa.

By 2030, we envision that strengthening the medical statistics capacity in Africa will be led by African institutions and medical statisticians, and our partnership-based approach will play an important role towards this vision.

## Data Availability

No new data were generated or analysed in support of this research.
